# Weaknesses in the Continuity of Care of Puerperal Women: An Integrative Literature Review

**DOI:** 10.1055/s-0043-1772185

**Published:** 2023-08-18

**Authors:** Ane Gabriele Poli Petersen, Adriane Cristina Bernat Kolankiewicz, Denise Casagrande, Pâmella Pluta, Vanessa Dalsasso Batista Winter, Fernanda Fernandes de Carvalho, Caroline Sissy Tronco

**Affiliations:** 1 Universidade Regional do Noroeste do Estado do Rio Grande do Sul, Ijuí, RS, Brazil

**Keywords:** transitional care, postpartum period, continuity of patient care, cuidado transicional, período pós-parto, continuidade da assistência ao paciente

## Abstract

The aim of the present study was to identify how the transition of care from the hospital to the community occurs from the perspective of puerperal women at risk. An integrative literature review was performed, with the question: “How does the transition of care for at-risk puerperal women from the hospital to the community occur?” The search period ranged from 2013 to 2020, in the following databases: PubMed, LILACS, SciELO, and Scopus. MESH, DeCS and Boolean operators “OR” and “AND” are used in the following crossover analysis: *patient transfer* OR *transition care* OR *continuity of patient care* OR *patient discharge* AND *postpartum period* , resulting in 6 articles. The findings denote discontinuity of care, given the frequency of non-adherence to the puerperal consultation. Transition studies of care in the puerperium were not found, which requires proposing new studies.

## Resumo

O objetivo do presente estudo foi identificar como ocorre a transição do cuidado do hospital para a comunidade na perspectiva de puérperas de risco. Foi realizada uma revisão integrativa da literatura, com a questão: “Como ocorre a transição do cuidado das puérperas de risco do hospital para a comunidade?” A pesquisa foi realizada com recorte temporal de 2013 a 2020, nas bases de dados: PubMed, LILACS, SciELO e Scopus. Utilizou-se MESH, DECS e operadores booleanos “OR” e “AND” resultando nos seguintes cruzamentos: *patient transfer* OR *transition care* OR *continuity of patient care* OR *patient discharge* AND *postpartum period* , com análise final de 6 artigos. Os achados denotam descontinuidade do cuidado, visto a frequência de não adesão à consulta puerperal. Estudos de transição do cuidado no puerpério não foram encontrados, o que requer que novos estudos sejam propostos.

## Introduction

Care transition (CT) is understood as a set of actions aimed at ensuring care continuity at different points of healthcare and or between different sectors of the same place. In addition, it encompasses a comprehensive care plan and should be performed by well-prepared professionals, whether in user and family education and their engagement as active subjects in decisions, or in the proper transfer of information between transition professionals. 1 In this context, maternity CT, after delivery, for primary health care (PHC), is a fundamental strategy.

The puerperium begins immediately after delivery, and lasts roughly 6 weeks, being its termination unforeseen, because it is related not only to anatomical and physiological changes, but to a framework of psychosocial issues that include self-esteem and reorganization of personal and family life. 2 Another author considers the variability of time that comprises this period, which can be from 8 months to 1 year. 3

On the other hand, the risk postpartum period is characterized by situations in which postpartum women present complications in their health condition, due to preexisting diseases or intercurrences generated by both organic and socioeconomic unfavorable factors. 4 In this period, women undergo physical, psychological, social, and cultural changes; thus, quality care aims to maintain maternal health and act early in the event of complications, to minimize or treat associated comorbidities. 5

Thus, aiming at the continuity of postpartum care, professionals and users/family members articulated in health services share information that contributes to the development of a care management plan both in the assistance provided in health services and in the promotion of supported self-care. From this, care continuity is the result of a joint, articulated, reflective, negotiated, singular and shared action. 6 In this perspective, inadequate CT can negatively affect treatment adherence, medication errors, low quality of life and increased risks for hospital readmissions. 7 8

In this context, the assistance to the puerperium covers several points of the Health Care Network (HCN), starting, first, in maternity, when the woman is oriented on care to her health, identification of warning signs and symptoms that indicate the need for reassessment in health services. 9 At this point of attention, it is recommended that qualified processes for the immediate postpartum be guaranteed, and the development of a care plan for the puerperal woman and the newborn (NB) and mechanisms of communication and integration with PHC for care continuity. 10

For this, the attention to the puerperium is complex and requires attention and continuity in the various points of attention of the HCN. The subject requires exploring the literature, and the development of the present study aims to identify how the transition from hospital care to the community occurs from the perspective of at-risk postpartum women.

## Methods

This is an integrative literature review study. The study consisted of six stages: 1) 1 ^st^ stage: identification of the theme and selection of the research question; 2 ^nd^ stage: establishment of inclusion and exclusion criteria; 3 ^rd^ stage: Identification of preselected and selected studies; 4 ^th^ Stage: Categorization of the selected studies; 5 ^th^ stage: Analysis and interpretation of the results, and 6 ^th^ stage: Presentation of the review/synthesis of knowledge. 11

The development was based on the following question: “How does the transition from the care of at-risk mothers from hospital to community occur?” For the construction of the research question, the PICO strategy (population, phenomenon of interest and context) was used, being possible, in this way, to elaborate a delimited and well-founded question that initiated the investigation. 12 The searches were based on Medical Subject Headings (MESH) and Health Sciences Descriptors (DeCS) and Boolean operators “OR” and “AND,” resulting in the following crossings: *patient transfer* OR *transition care* OR *continuity of patient care* AND *charge* OR *patient discharge* AND *postpartum period* .

The selection of articles took place from June 2021 to June 2022, in the following databases: National Library of Medicine National Institutes of Health (PubMed), Latin American and Caribbean Health Sciences Literature (LILACS), Scientific Electronic Library Online (Scielo) and SciVerse Scopus (Scopus).

The process of searching and analyzing the studies was performed jointly by four researchers, aiming at data reliability. The inclusion criteria were: primary studies, in Portuguese, English and Spanish, in the period from 2013 to 2020, moment when the World Health Organization (WHO) launches the 2 ^nd^ update of guidelines for postnatal care focusing on mothers and newborns in countries with limited resources, with the aim of reducing maternal and neonatal deaths from the review of evidence-based best practices. 13 The exclusion criteria were: review studies, theses, dissertations, editorials, case studies, and manuals. 11

**Fig. 1:**
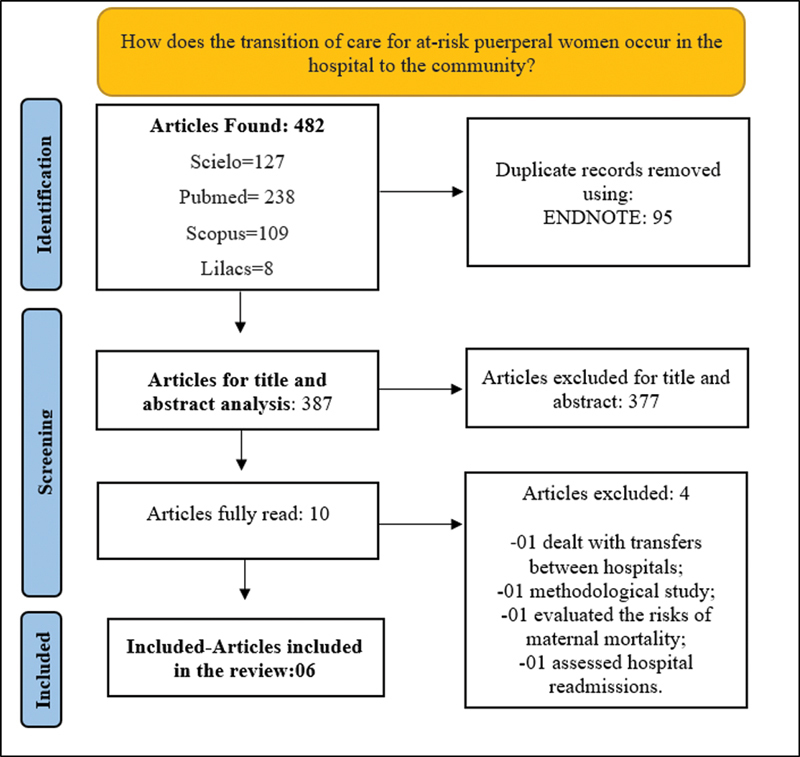
PRISMA Flowchart.

The searches in the aforementioned databases resulted in 482 studies, 127 from SciELO, 238 from PubMed, 109 from Scopus, and 8 from LILACS. Thus, to organize the references found, an online tool called ENDNOTE was used, which allowed, mainly, the identification of duplicate articles. After the exclusion of duplicate articles, the titles and abstracts of the 387 remaining articles were read, of which 10 met the inclusion criteria and were fully read. The reading was performed separately by the four researchers and the disagreements in the analysis were discussed in a meeting of experts. Thus, eight studies were part of the corpus of the research ( Fig. 1 ).

Given the extraction of data from the results, an instrument adapted from the literature by the authors was used, which includes characteristics of article identification (title, authors, year, country of publication, type of study, main contributions). The results were arranged through charts and the analysis of articles was performed descriptively, with the synthesis of evidence from each publication.

## Results

Of the six studies included, five belonged to the PubMed database and one to the SciELO database. Of these, five articles are in English and one in Portuguese. According to the years of publication, two were published in 2016, one in 2017, and three in 2020. The countries of origin were: Australia, Turkey, United States of America, Ethiopia, Sweden, and Brazil, with one article each. The number of participants was 22,777 women. The studies are described and listed in Chart 1 .

**Chart 1:** Presentation of included studies

First Author, Year, Country and Title	Type of study and number of participants	Contribution of the study
1. WOODWARD, B.M. 2016. Australia. Beyond birth: Women's concerns about post-birth care in an Australian urban community. 14	Qualitative study, through semi-structured interviews. 15 participants.	There is a gap in the postpartum period characterized by a lack of information and psychosocial care, both in the public and private sectors, to guarantee the continuity of care. The highest levels of satisfaction occurred in women who opted for home birth.
2. BARIMANI, M. 2016. Sweden. Support and continuity during the first 2 weeks postpartum. 15	Cross-sectional study, with mixed methodology. 363 participants.	Part of the puerperal women pointed out the lack of continuity, the insufficient support in relation to the physical and emotional health of the women. The level of satisfaction was related to the time the women stayed in the hospital, that is, they were given direct postpartum care. Mothers who sought emergency care showed lower satisfaction with puerperal care. The assistance sought in the emergency triggered the need for support and continuity of care.
3. YANIKKEREM, E. 2017. Turkey. Factors affecting readiness for discharge and perceived social support after childbirth. 16	Descriptive and cross-sectional method, cross-sectional study. 610 participants.	The information provided by professionals about postpartum care and social support is important for women and their families. Especially for the primiparous group, women who had complications during or after childbirth or who for some reason could not attend childbirth classes. Women in socially vulnerable conditions have less information about the postpartum period and were not prepared for hospital discharge. There is a need for special intervention programs that inform this population about postpartum care
4. WEN, T. 2020. United States. Fragmentation of postpartum readmissions in the United States. 17	Cohort study, retrospective 21,789 participants.	Reducing care fragmentation can represent an important objective to improve postpartum care and reduce the risk of severe morbidity, high costs, and long hospital stays.
5. ASRATIE, M.H. 2020. Ethiopia. Completion of maternity continuum of care among in the post-partum period: Magnitude and associated factors in the north west, Ethiopia. 18	Cross-sectional study 819 participants	Fragility in the continuity of maternity care, with less than half of the women having a puerperal consultation. As associated factors, we identified the fact that the majority live in rural areas and low schooling.
6. BITTENCOURT, S.D.A. 2020. Brazil. *Nascer no Brasil* : continuity of care during pregnancy and postpartum period for women and newborns. 19	Cross-sectional, quantitative study 16,220 participants.	Only 32.2% of the study population had a postpartum consultation. The coordination of care is still a challenge in the health care of women and children in the pregnancy-puerperal period, especially in the north and northeast states.

The first study used a qualitative method through semistructured interviews, and sought to know the perceptions of women about forms of postpartum care. It was held in Australia and involved 15 women. The study highlighted an important gap in the postpartum period characterized by lack of information and psychosocial care, both in the public and private sectors. However, it still showed that women who gave birth in the public sector had a better follow-up, all received home visits or a phone call within 10 days and a guarantee that they had someone to examine them and take care of them. The highest levels of satisfaction were women who opted for home birth, who had a midwifery follow-up in the prenatal, childbirth and postpartum periods and did not report concern or insecurity.

From this perspective, the second study adopted a mixed methodology; it was conducted in Stockholm, Sweden, and involved 363 women. It investigated the satisfaction perceived by mothers regarding prenatal care, postpartum care, and child healthcare in the first two postpartum weeks. Child and postpartum health support were considered equally satisfactory, while prenatal support was classified as much less satisfactory. The chances of being satisfied with postpartum support were twice as high for mothers who did not have emergency consultations after delivery when compared with those who had emergency visits. Mothers who remained in the hospital for ≥ 3 days were 3 times more satisfied with postpartum support compared with those who remained for 2 days.

The third study was conducted in Turkey in 2015 and aimed to assess readiness for hospital discharge and the perception of social support received in the postpartum period. It involved 610 participants and used an instrument divided into three parts, Readiness for Hospital Discharge Scale – New Mother Form (RHD-NMF), which was developed by Weiss et al. 20 that evaluates whether the woman is ready to be discharged from hospital and the third part included the “Multidimensional Scale of Perceived Social Support” (MSPSS) that was developed by Zimmet in 1988 to assess social support. Most women (94.3%) reported being ready to go home. In this sense, evaluating the factors that lead to readiness for hospital discharge, it was found that 85.9% received information about hospital discharge and that most of these were provided by doctors and nurses.

Regarding the fourth study, this was a retrospective cohort study, conducted in the United States, from 2010 to 2014, with 21,789 participants. This study aimed to characterize the risk and results associated with postpartum fragmentation in readmissions where the readmission hospital was different from the delivery hospital. As a result, evaluating the indications for readmission, fragmentation was more likely for heart failure (28.6%), thromboembolism (28.4%), and respiratory infections (33.9%). Less likely causes include hypertension (11.1%), wound complications (10.7%), and uterine infections (11.0%). Thus, it was concluded that discontinuity of postpartum care was associated with increased risk of severe morbidity.

The fifth study, in turn, was conducted in the city of Motta (northwest Ethiopia, Africa) in 2019. At the time, it sought to evaluate the completion of continuing maternity care, the Continuum of maternity care, that is, the continuation of care from pregnancy to the postpartum period. In this study, 77.4% of women lived in rural areas, 69.6% without formal education and 63% started prenatal consultations in the 2 ^nd^ trimester of pregnancy. Of the 819 participants, 283 had home births and only 10 received postpartum care by health professionals. Overall, of the 819 women, 346 (47%) had postpartum appointments.

The sixth study was conducted in Brazil, from 2011 to 2012, consisting of a cross-sectional and quantitative study that aimed to estimate the adequacy of the healthcare line during pregnancy and postpartum in puerperal and newborn users of the Brazilian Unified Health System (SUS, in the Portuguese acronym). Of this, 16,220 women participated. It was found that the southeastern (41.7%) and northeastern (29.4%) regions concentrated most of the births. Of the total participants, 74.8% of the women started prenatal care until the 16 ^th^ week of pregnancy and only 32.2% underwent the puerperal consultation. The study revealed that there is a lower chance of care continuity in women living in the northeast, north, and midwest regions. In multivariate analysis, considering schooling, parity, and place of residence, the North and Northeast regions presented a seven to ten times greater chance of inadequate care than the South region. These findings indicate that the coordination of care is still a challenge in the healthcare of women and children in the puerperal pregnancy period.

## Discussion

Of the six studies included, two showed more subjective issues involving women's perception and satisfaction in the postpartum period; one addressed readiness for hospital discharge, one involved readmission issues in the postpartum period and associated risk factors; a study on the *continuum* of maternity care, and finally, a study involving the adequacy of the maternal-child care line.

The currently available evidence does not address hospital CT for the community of puerperal women, nor those considered at risk. In short, all studies mentioned directly or indirectly care fragmentation, the fragility of the *continuum* of maternal care evidenced the lack of puerperal consultation and the inadequacy of the line of maternal and childcare. Another study points out that care actions are incipient, and that comprehensive care is expected in the RAS, it is fragile, also considering the lack of follow-up after hospital discharge. 21 Therefore, the CT process from the hospital to home is a challenging moment, as sometimes it is necessary for this care to be performed by the family members themselves. 22

Study 1, conducted in Australia, found a gap in the postpartum period characterized by lack of information and psychosocial care. Still, women assisted in the public sector had better postpartum follow-up compared with the private sector. As for the guidance on reevaluation in health services, study 2, Sweden, made clear that the most sought service are the emergency sectors, denoting disarticulation with PHC. In terms of satisfaction perceived by women, there is greater satisfaction of those who opted for home birth, study 1, and who had a continuous follow-up in the prenatal, childbirth, and puerperium periods. Satisfaction was also perceived in women, study 2, who remained longer in hospital, that is, who had more direct and prolonged postpartum care.

For Barimani et al, 15 greater flexibility in hospital stay could improve the satisfaction of new mothers. However, understanding that satisfaction is related to more direct assistance in the postpartum period, it can be inferred that care continuity needs to be guaranteed in PHC, since it is responsible for coordination of care and longitudinal monitoring. 10 A systematic review study with meta-analysis showed a significant reduction of one and a half days in hospital stay in favor of patients who received CT intervention. 23

Therefore, for the care continuity to occur, it is recommended that the maternity hospital report the discharge of the puerperal woman and the NB to the health unit of the PHC to which they are linked so that the appointments are scheduled, guaranteed the First Week of Integral Health (4). Regarding readiness for hospital discharge, the original study that validated the Readiness for Hospital Discharge Scale (RHDS) was published in 2006 and involved 356 participants, including 121 adult patients (medical-surgical), 122 postpartum mothers and 113 fathers of hospitalized children. 20 In Brazil, the instrument for adults was adapted transculturally in 2015; 24 however, the specific version of the puerperal women has not been validated so far. This version is only available in English, Chinese, Spanish, Turkish, and Polish. 25

In study 3, from Turkey, the RHDS scale was applied for puerperal women, showing that 94.3% were ready for hospital discharge and 85.9% received guidance for discharge by doctors and nurses. The scale was also used in Poland, study 4, and data indicated that 96.5% of women reported being ready for discharge. The perception of women regarding readiness for hospital discharge is related to their participation in the discharge process. From this evaluation, it is possible to identify early mothers at risk of problems in the postdischarge period, especially those who need more care and monitoring, to prevent adverse results. 26

Regarding readmissions and readmissions in the postpartum period, a study performed in Tunisia (North Africa) had as statistically significant risk factors for readmission cesarean section, emergency cesarean section, anemia, and thrombocytopenia. 27 A study conducted in Massachusetts, United States, confirms the finding of increased risk of readmissions after cesarean delivery. It also cites as main causes complications of the surgical wound and infections. 28

Which sought to relate the causes of hospital readmissions associated with fragmentation of care in the postpartum period, also found complications of the wound and uterine infections as associated causes. It also related these findings to high hospital costs and long duration of hospitalizations. In addition to the causes of readmissions identified, women may present an increased risk of certain morbidities in their subsequent pregnancies. 29 Another study that aimed to analyze puerperal complications identified a high prevalence of complications associated with the high rate of cesarean sections and invasive procedures in vaginal delivery. 30 Therefore, it is expected that, after hospital discharge, this care will be continuous, to provide comprehensive care, by the multidisciplinary team of primary healthcare. 21

Concerning the continuing care from maternity to the community, the two studies that addressed this theme showed that the puerperal consultation occurred in 47% in Ethiopia (Africa) and 32.2% in Brazil. In this context, Brazilian studies have identified a low rate of adherence to puerperal consultation, ranging from 16.8 to 43.08%. 31 32

The studies mentioned above consider adherence to puerperal consultation as, at least, the attendance to one consultation. Nevertheless, this scenario falls short of that recommended by the WHO, 33 which provides for a minimum of 3 postpartum consultations, thus distributed: one between 48 and 72 hours after delivery; another between 7 and 14 days, and the third, in the 6 ^th^ week.

When the reasons for nonadherence are investigated, there are reasons for forgetfulness, complications with the NB, transportation difficulties, and distance between home and health unit. 34 As associated factors, it is found that puerperal consultation is related to lower income and schooling, as well as not being addressed/valued during prenatal consultations. 35

In this regard, to investigate the orientation on the importance of puerperal consultations both during prenatal care and in the immediate postpartum period in the hospital, Vilela et al. 36 performed a study with 216 puerperal women, in a municipality of the state of Mato Grosso, Brazil. They found that 92.1% of the puerperal women did not receive prenatal care and only 5.6% were guided at the hospital. Thus, it shows the need for measures that promote the awareness of health professionals about the importance of guidance as well as the scheduling of puerperal consultation, effectively effecting the referral and counter-referral system.

## Conclusion

The present study showed the scarcity of research related to continuity of care from the perspective of postpartum women, which unveils a knowledge gap, requiring greater emphasis and concern with this theme, to improve the care of this population. It was possible to identify weaknesses in the postpartum care, both in relation to the guidelines provided as to the care issues offered in the hospital and in primary health care. Cesarean section was identified as a risk factor for hospital readmission. The mothers' perceived satisfaction was related to a continuous follow-up in the pregnancy-puerperal cycle. However, the findings show discontinuity of care, given the frequency of nonadherence to puerperal consultation. Although readiness for discharge has identified adequate rates, there are few studies in this area, since the RHDS instrument for postpartum women is validated in few countries. Transition studies of postpartum care were not found, which requires proposing new studies.
